# 749. A Nurse-Driven Protocol for Early Detection of Clostridiodes difficile Infections

**DOI:** 10.1093/ofid/ofab466.946

**Published:** 2021-12-04

**Authors:** Shannon Beckman, Jonathan Chia, Bethany Stibbe, Monica Rykse, Michael S Wang

**Affiliations:** 1 Spectrum Health, Saint Joseph, Michigan; 2 Spectrum Health Lakeland, St Joseph, Michigan

## Abstract

**Background:**

*Clostridiodes difficile* infections (CDI) are a significant cause of hospital acquired infections, resulting in significant morbidity and mortality. Early detection of CDI has been shown to reduce the spread of CDI within the hospital. As nurses are frequently at the patient’s bedside, we proposed to empower the nursing staff to assess, collect stool samples, and order *C. difficile* testing.

**Methods:**

Rates of CDI were measured by our Infection Control Department. Hospital-onset CDI (HO-CDI) was defined as a positive *C. difficile* PCR assay after 3 days of admission, defined as a stay of at least 3 midnights. Community-onset CDI (CO-CDI) was defined as any case that was diagnosed in the Emergency Department or inpatient ward < 3 days of hospitalization based on stool testing as above. Nursing was instructed and empowered to assess, collect stool specimens, and place an order for *C. difficile* testing, based on the criteria of ≥3 loose or watery stools over 24 hours. Nursing was also educated to not order a test if patients had received stool softeners, enemas, or laxatives within 24 hours. The protocol was initiated in February 2019.

**Results:**

Rates of HO-CDI increased during the intervention period, rising from 2.6 cases/10000 patient days and peaking at 17.7 cases/10000 patient days (average 6.7 vs. 12.1 monthly cases per 10,000 patient days. Rates of CO-CDI did not significantly change (12.4 vs. 11.5 monthly cases per 10000 patient days). Due to concerns of inappropriate testing, which included testing after laxatives, enemas, or sending specimens despite < 3 stools over 24 hours, the protocol was discontinued in June 2019. Although the HO-CDI rate remained elevated over the next month, the rate subsequently decreased over the next several months (12.1 vs. 8.0 cases per 10000 patient days). Overall testing also increased over the study period (148.3 vs. 169.9 cases/per 10000 patient days).Figure 1 - Clostridiodes difficile rates

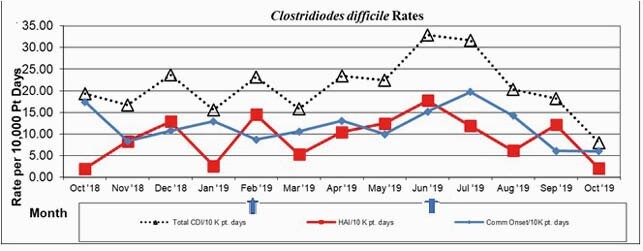

Figure 2 - CDI testing rates

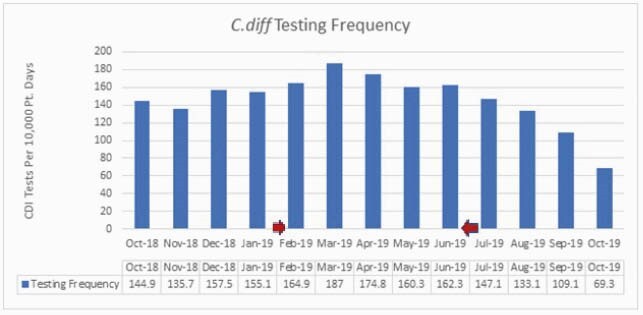

**Conclusion:**

A nursing driven protocol resulted in increased HO-CDI and overall CDI rates suggesting that the intervention may have been a factor in increasing the frequency of HO-CDI diagnoses, although the possibility of misdiagnosis of colonization for true CDI cannot be excluded. Further education of nursing staff may be a potential intervention in improving appropriate CDI testing.

**Disclosures:**

**All Authors**: No reported disclosures

